# Suspended Development in Twin Pregnancies

**Published:** 1867-04

**Authors:** M. M. Eaton

**Affiliations:** Peoria, Ill.


ARTICLE XVIII.

### SUSPENDED DEVELOPMENT IN TWIN PREGNANCIES.

By M. M. EATON, M.D., Peoria, Ill.

In the *May No.* of the *Chicago Medical Journal* for 1864, Vol. 21, No. 5, page 206, I reported a case that I attended, where a lady was delivered of a fœtus of about two months development, about two hours previous to her being delivered of a still-born fully developed child, at full term.

The past summer, having had another case of a similar nature, I thought I would report this also, simply to add an item to accumulating experience on the physiology and pathology of gestation; such cases being of special interest to the pathologist, and not entirely void of interest to the general practitioner.

CASE II. Was called, August 14, 1866, to attend Mrs. H., living on North A—— St. Found her to be about 21 years of age, native of Illinois, in labor with her first child. She stated that she had always enjoyed good health, and she had the appearance of being in perfect health. On making an examination, I discovered the head of the child presenting to the os uteri, which was dilated about 2½ inches, with a gristly substance hanging from the os, an inch or more in length. This, by more careful manipulation, I discovered to be the hand and arm of a small fœtus. I then made slight traction in the absence of the pain, and the fœtus was delivered. It was of the size of a three months fœtus, was not decomposed. The funis consisted simply of a slender membrane, without any appearance of bloodvessels. I should state that the amniotic fluid had been previously discharged. The labor now progressed naturally, and a fully developed living girl was delivered within an hour. The mother recovered without any trouble; the child still lives, a stout and hearty child.

Upon delivering the placenta, I could discover no indication of the attachment of the funis of the fœtus to the placental surface. I have these two specimens of arrested development carefully preserved in alcohol. Now, the question arises, what caused the suspension of development in one and not in the other of these twins?

